# Soluble expression of an amebic cysteine protease in the cytoplasm of *Escherichia coli* SHuffle Express cells and purification of active enzyme

**DOI:** 10.1186/s12896-018-0429-y

**Published:** 2018-04-03

**Authors:** Ekaterina Jalomo-Khayrova, Rosa E. Mares, Patricia L. A. Muñoz, Samuel G. Meléndez-López, Ignacio A. Rivero, Marco A. Ramos

**Affiliations:** 1 Facultad de Ciencias Químicas e Ingeniería, Universidad Autónoma de Baja California, Calzada Universidad 14418, Parque Industrial Internacional, 22390 Tijuana, BCN México; 2 Centro de Graduados e Investigación en Química, Instituto Tecnológico de Tijuana, Boulevard Industrial S/N, Mesa de Otay, 22510 Tijuana, BCN México

**Keywords:** Recombinant protein, Amebic cysteine protease, Expression and purification, Cytosolic oxidative folding, Proteolytic activity, Enzyme inhibition

## Abstract

**Background:**

Recombinant production of amebic cysteine proteases using *Escherichia coli* cells as the bacterial system has become a challenging effort, with protein insolubility being the most common issue. Since many of these enzymes need a native conformation stabilized by disulfide bonds, an elaborate process of oxidative folding is usually demanded to get a functional protein. The cytoplasm of *E. coli* SHuffle Express cells owns an enhanced ability to properly fold proteins with disulfide bonds. Because of this cellular feature, it was possible to assume that this strain represents a reliable expression system and worthwhile been considered as an efficient bacterial host for the recombinant production of amebic cysteine proteases.

**Results:**

Using *E. coli* SHuffle Express cells as the bacterial system, we efficiently produce soluble recombinant *Eh*CP1protein. Enzymatic and inhibition analyses revealed that it exhibits proper catalytic abilities, proceeds effectively over the substrate (following an apparent Michaelis-Menten kinetics), and displays a typical inhibition profile.

**Conclusions:**

We report the first feasibility study of the recombinant production of amebic cysteine proteases using *E. coli* SHuffle Express as the bacterial host. We present a simple protocol for the recombinant expression and purification of fully soluble and active *Eh*CP1 enzyme. We confirm the suitability of recombinant *Eh*CP1 as a therapeutic target. We propose an approachable bacterial system for the recombinant production of amebic proteins, particularly for those with a need for proper oxidative folding.

**Electronic supplementary material:**

The online version of this article (10.1186/s12896-018-0429-y) contains supplementary material, which is available to authorized users.

## Background

Proteases are hydrolytic enzymes that cleave the peptide bond that joins amino acids in proteins [1, 2]. Based on the cleavage site at the substrate, they are usually subdivided into exopeptidases and endopeptidases [1, 3]. However, according to their catalytic mechanism and the amino acid residue present at the active site, are so grouped in aspartic proteases, cysteine proteases, glutamic proteases, metalloproteases, asparagine proteases, serine proteases, threonine proteases, and proteases with mixed or unknown catalytic mechanism [4]. Because of their biochemical function play significant roles in several processes that are critical for cell life, such as degradation of potentially toxic misfolded or damaged polypeptides [5].

Worldwide, protozoan parasites infectious to humans represent a major threat to public health [6, 7]. Intracellular parasites, such as *Toxoplasma*, *Leishmania*, *Plasmodium*, and *Trypanosoma*, are among the most lethal [8]. *Cryptosporidium*, *Entamoeba*, and *Giardia* infect the gastrointestinal tract causing diarrhea, which is fatal if left untreated [9], while *Trichomonas* colonizes the epithelium of the urogenital tract producing inflammation in the cervix, vagina, and urethra [10]. These pathogens encode a variety of proteases involved in essentially all aspects of their biology, including (i) cell invasion, development, and migration, (ii) evasion of host immune system, and (iii) degradation of proteins for nutrition [11, 12]. Thus, these proteolytic enzymes have medical and pharmaceutical importance as they are valuable targets for designing novel or improved therapeutic compounds [3, 6–10, 13–17].

*Entamoeba histolytica* (*Eh*), the causative agent of human amebiasis [18], has 86 genes encoding proteolytic enzymes: 50 cysteine proteases, 22 metalloproteases, 10 serine peptidases, and 4 aspartic proteases [19]. Of these, secreted cysteine proteases (CP) play significant roles in pathogenicity, i.e., proteolytic degradation of host extracellular matrix components [20, 21]. Different studies have shown that the high expression levels of three enzymes: *Eh*CP1, *Eh*CP2, and *Eh*CP5, account for roughly 90% of the CP-specific activity [22, 23]. Interestingly, *Eh*CP1 is unique to *E. histolytica*, since the orthologous gene is absent in *E. dispar* [22, 24], a morphologically undistinguishable but non-pathogenic ameba [25]. As an inactive precursor or zymogen, which comprises a pro-domain that blocks the active site, depends on the cleavage of the signal peptide and the limited proteolysis of the pro-domain to get its mature form (Additional file 1: Figure S1).

To date, several recombinant approaches have been undertaken to produce *Eh*CP proteins using *Escherichia coli* cells as the bacterial system [24, 26–29]. Although active enzymes were satisfactorily obtained, protein insolubility was the major challenge found, requiring an elaborate oxidative protein folding process to promote solubility. *E. coli* SHuffle Express, a mutant strain recently been developed and successfully proved to own an enhanced ability to correctly fold proteins with disulfide bonds in the cytoplasmic compartment [30]. Considering the foregoing, we took a chance on this bacterial system for soluble expression of recombinant *Eh*CP proteins. Here, we present efficient recombinant production of fully soluble and active *Eh*CP1 enzyme using *E. coli* SHuffle Express strain as the bacterial host.

## Results

First, using pQE30 as the parental vector, the recombinant pQEhCP1 plasmid (Additional file 1: Figure S2) was successfully constructed by subcloning the gene fragment encoding the pro-mature *Eh*CP1 protein under the control of the T5/lacO (promoter-operator) sequence. Competent *E. coli* SHuffle Express cells were efficiently transformed with pQEhCP1 and recombinant protein was easily induced with 0.5 mM isopropyl-β-D-1-thiogalactopyranoside (IPTG). After bacterial culturing for 18 h at 30 °C, recombinant *Eh*CP1 protein tagged with a hexahistidine sequence at the N-terminus was readily purified by two consecutive chromatographic protocols: nickel-affinity and gel permeation. Analytical expression assays revealed proper gelatinase activity (Additional file 1: Figure S3). Furthermore, quantitative analysis of the expression and purification process showed a reasonable outcome in terms protein purity and protease activity (Table 1).

**Table 1:** Purification of recombinant *Eh*CP1 (summary)

Purification Step	Total Protein (mg) ^b^	Total Activity (units) ^c^	Specific Activity (units/mg) ^c^	Recovery Yield (%) ^d^	Enzyme Purity (%) ^e^
Crude lysate ^a^	69.75	9918.5	142.2	100.0	2.4
Nickel-affinity	0.81	4731.2	5841.0	47.7	97.0
Gel permeation	0.65	3915.0	6023.0	39.5	100.0

^a^Obtained from five cell pellets, each from a 200-mL batch (total: 1 L of bacterial culture). ^b^Protein concentration determined by Bradford assay. ^c^Protease activity measured by the chromogenic assay. ^d^Relative amount of total activity at each step as compared to the first. ^e^Relative amount of specific activity at each step as compared to the last

**Fig. 1:**
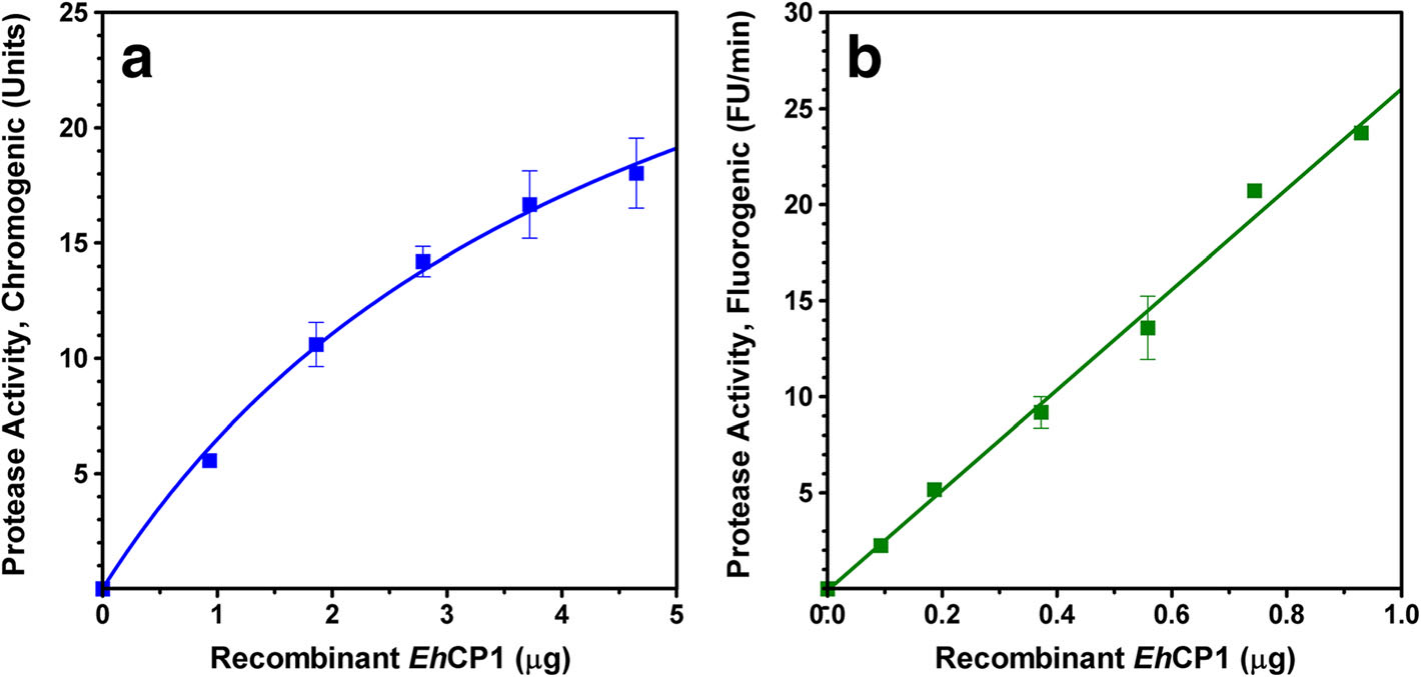
Effect of the enzyme concentration on the rate of reaction. **a** Hydrolysis of Z-Arg-Arg-pNA by recombinant *Eh*CP1 (0–4.65 μg). **b** Hydrolysis of Z-Arg-Arg-AMC by recombinant *Eh*CP1 (0–0.93 μg). Data represent the mean ± S.E.M. (bars) of 2–3 independent experiments

**Fig. 2:**
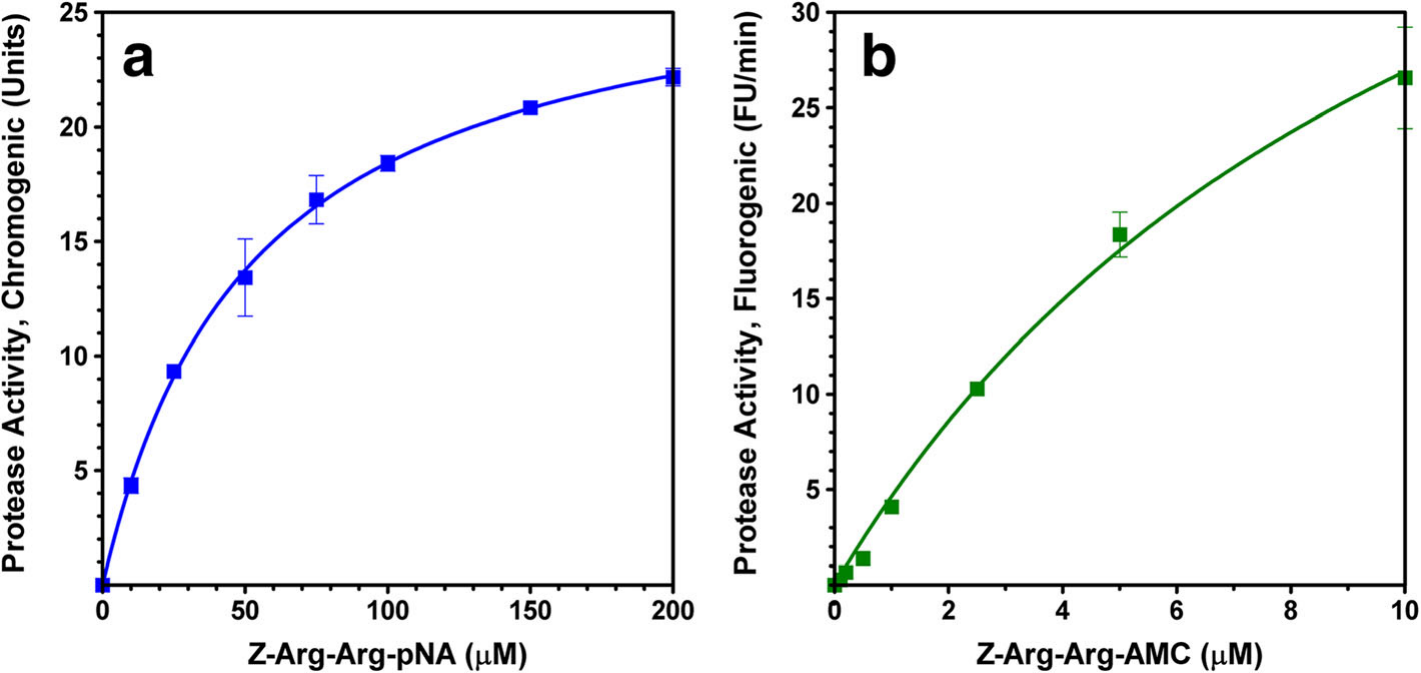
Effect of the substrate concentration on the rate of reaction. **a** Hydrolysis of Z-Arg-Arg-pNA (0–200 μM) by recombinant *Eh*CP1 (0.05 mg/mL). **b** Hydrolysis of Z-Arg-Arg-AMC (0–10 μM) by recombinant *Eh*CP1 (0.005 mg/mL). Data represent the mean ± S.E.M. (bars) of 3 independent experiments

Protease activity of recombinant *Eh*CP1 was effectively measured using two synthetic peptides as substrates, Z-Arg-Arg-pNA (chromogenic) and Z-Arg-Arg-AMC (fluorogenic). While considering the pH value of 6.5 as standard (as maximal activity was found within 5.5 and 7.5 [24]), the assay temperature was simply decided from preliminary measurements (using the initial rate as a test indicator): 37 °C for chromogenic and 22 ± 1 °C for fluorogenic. Since the precise amide bond at the substrate was efficiently recognized and hydrolyzed by enzymatic action, each released product (p-nitroaniline, pNA; 7-amino-4-methylcoumarine, AMC) was correspondingly monitored and used to calculate the protease activity. Depicted in Fig. 1, the enzyme concentration positively affects the rate of reaction, i.e., an increase in the recombinant *Eh*CP1 amount improves the protease activity. Likewise, the substrate concentration stimulates the rate of reaction, and thus the protease activity (Fig. 2). Thoroughly examining the latter, using enzyme at 0.05 mg/mL (for chromogenic assay) or 0.005 mg/mL (for fluorogenic assay), we obtained sound kinetic parameters for the hydrolysis of both substrates: Z-Arg-Arg-pNA (Km = 57.1 μM; Vmax = 29.3 units; SA = 5856.6 units/mg) and Z-Arg-Arg-AMC (Km = 6.7 μM; Vmax = 19 FU/min; SA = 38,088 FU/min/mg).

**Fig. 3:**
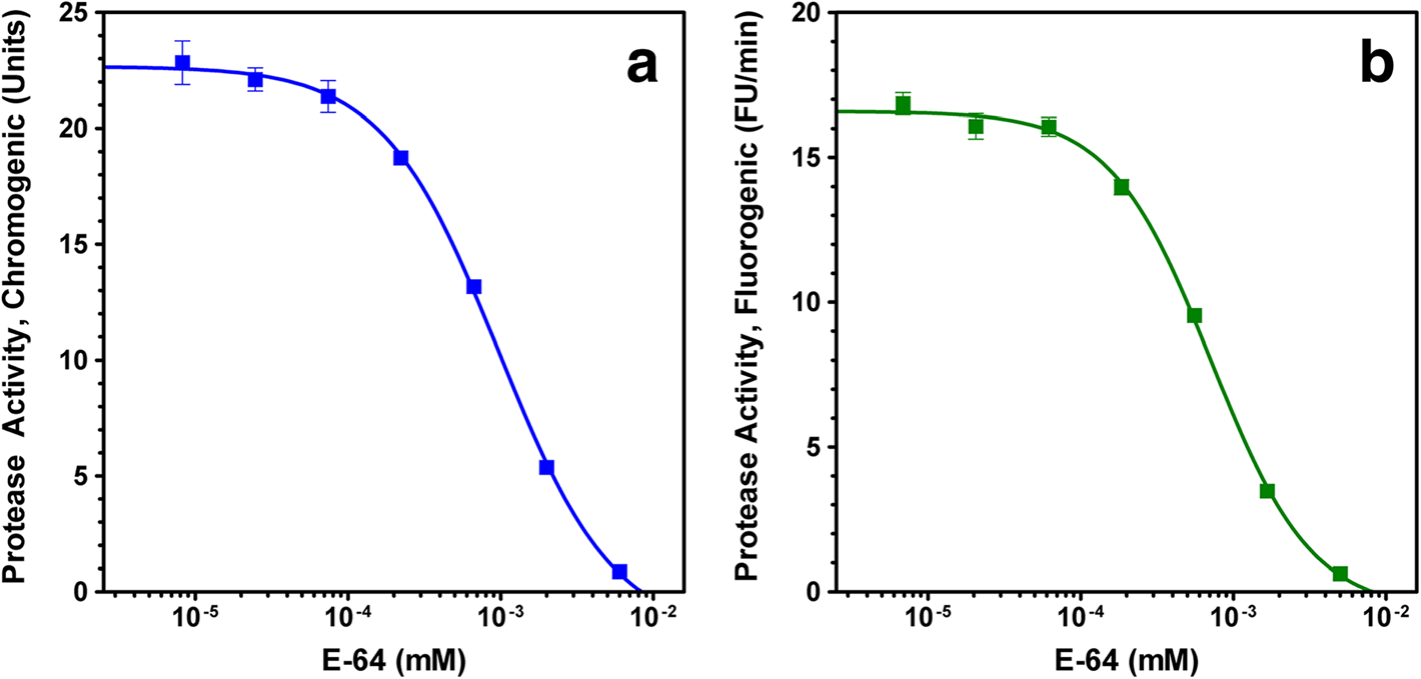
Effect of the E-64 concentration on the protease activity of recombinant *Eh*CP1. The enzyme and inhibitor (0–6 μM) interact for 15 min before the assay. Hydrolysis of 200 μM Z-Arg-Arg-pNA (**a**) and 10 μM Z-Arg-Arg-AMC (**b**) by recombinant *Eh*CP1 (as in Fig. 2). Data represent the mean ± S.E.M. (bars) of 3 independent experiments

Proven so far, recombinant *Eh*CP1 exhibits specific catalytic abilities, proceeds effectively on the substrate, and follows an apparent Michaelis-Menten kinetics. Finally, to assert its value as a therapeutic target, the effect of L-*trans*-3-carboxyoxiran-2-carbonyl-L-leucylagmatine (E-64) on the protease activity was further evaluated. E-64 is a well-known CP-specific inhibitor that functions as an effective active-site titrant [31]. Displayed in Fig. 3, the sigmoidal curve indicates that inhibition of protease activity follows a typical dose-response profile. Also, using the enzyme concentration as before (for kinetic parameters), we found the IC_50_ at 1 μM.

## Discussion

We used *E. coli* SHuffle Express cells harboring a recombinant plasmid to produce *Eh*CP1 protein. Along with the mature domain, the recombinant enzyme comprises the pro-domain, which is necessary for preserving its inactive state and apparently required for proper folding [28]. As compared to those reported producing an active thioredoxin-tagged protein (Trx-*Eh*CP1) [24]: insoluble material (about 10 mg/L) that requires optimal refolding conditions to get reliable enzymatic activity, our approach represents progress in the recombinant production of soluble and active *Eh*CP1 protein. Even so, since a limited yield of pure protein was found (0.65 mg/L), we acknowledge the need for further studies to improve production levels. Despite the apparent dissimilarity between the calculated Km values for two synthetic peptides used as substrates, Z-Arg-Arg-pNA and Z-Arg-Arg-AMC, which was attributed to the chemical nature of the leaving group (pNA or AMC) [32], the observed value for hydrolysis of Z-Arg-Arg-AMC (6.7 μM) closely resembles those reported using other recombinant enzymes, such as Trx-*Eh*CP1 (2 μM) [24] and *Eh*CP5 (7.29 μM) [33]. Also, as a protease sensitive to E-64 (a CP-specific inhibitor), it exhibits an IC_50_ value (1 μM) comparable to that established for recombinant *Eh*CP5 (6 μM) [33]. Altogether, it is fair to presume suitability of the recombinant *Eh*CP1 as a therapeutic target.

## Conclusions

Search for new or improved compounds with therapeutic activity requires the identification and validation of targets (i.e., proteins with the potential of being blocked, inhibited, modulated or regulated, when interacting with a drug) [34]. The availability of genomic data has notably contributed towards distinguishing putative targets [35, 36]. Since isolation and purification of proteins from their source is often a challenging task, the recombinant production using an efficient expression system represents a promising approach to get suitable material (i.e., pure and functional proteins) [36, 37].

In this study, we report the recombinant production of *Eh*CP1 using *E. coli* SHuffle Express strain as the bacterial host. We present a simple protocol for the expression and purification of the recombinant protein as a soluble and active enzyme. Through the analysis of protease activity and enzymatic inhibition, we confirm its reliability as a therapeutic target. Finally, we propose an approachable bacterial cell system for the recombinant production of other amebic proteins, particularly those with a need for proper oxidative folding.

## Methods

### Materials

DNA amplification reagents and purification kits were acquired from Qiagen (Germantown, MD, USA). Bacterial culture medium components were obtained from Becton, Dickinson and Company (Franklin Lakes, NJ, USA). Protein analysis and purification reagents were supplied by Bio-Rad Laboratories (Hercules, CA, USA), GE Healthcare Bio-Sciences (Pittsburgh, PA, USA), and Qiagen. Endonucleases and other enzymes were available from New England Biolabs (Ipswich, MA, USA). Unless otherwise mentioned, additional reagents were provided by Sigma-Aldrich (St. Louis, MO, USA). All materials were biochemical or biotechnological research grade.

### Bacterial strain and plasmids

The *Escherichia coli* strains and plasmids used in this study are listed in Table 2. Bacterial cultures were grown in LB medium (10 g/L tryptone; 5 g/L yeast extract; 10 g/L NaCl) supplemented with ampicillin (0.15 mg/mL). Xl1-Blue MRF’ was used as the host for molecular cloning, while SHuffle Express for protein expression.

**Table 2:** *E. coli* strains and plasmids

Strains or Plasmids	Relevant Genotype or Features	Source
Strains		
XL1-Blue MRF′	*Δ(mcrA)183 Δ(mcrCB-hsdSMR-mrr)173 endA1 supE44 thi-1 recA1 gyrA96 relA1 lac [F′ proAB lacI* ^*q*^ *ZΔM15 Tn10 (Tet* ^*R*^ *)]*	Stratagene ^a^
SHuffle Express	*fhuA2 [lon] ompT ahpC gal λatt::pNEB3-r1-cDsbC (Spec* ^*R*^ *, lacI* ^*q*^ *) ΔtrxB sulA11 R(mcr-73::miniTn10–Tet* ^*S*^ *)2 [dcm] R(zgb-210::Tn10 –Tet* ^*S*^ *) endA1 Δgor ∆(mcrC-mrr)114::IS10*	New England Biolabs
Plasmids		
pQE30	Lactose regulation, ColE1 origin, Amp^R^	Qiagen
pQEhCP1	pQE30-based, recombinant *Eh*CP1	This study

^a^Agilent Technologies (Santa Clara, CA, USA)

### Construction of pQEhCP1

The plasmid expressing recombinant *Eh*CP1 was obtained by subcloning the gene fragment encoding the pro-mature polypeptide (UniProt Q01957, Ile^14^ to Leu^315^) into the commercially available vector pQE30. Molecular cloning was performed according to standard protocols [38]. The gene fragment was amplified by PCR from genomic DNA of *E. histolytica* (HM1:IMSS strain) using the synthetic primers PROEHCP1F (5′-gcg gat cca ttg att tca ata cat ggg ttg cca ata ac-3′) and PROEHCP1R (5′-cga agc ttt cag aga tat tca aca cca gtt gga taa ag-3′), which were designed to include the BamHI and HindIII restriction sites at the 5′ and 3′ ends, respectively. After endonucleolytic digestion, the PCR product was inserted into pQE30. The recombinant plasmid (pQEhCP1) was extracted from transformed XL1-MRF’ cells and characterized by an astringent endonucleolytic analysis. The authenticity of the insert was confirmed by DNA sequencing.

### Expression of recombinant *Eh*CP1

The recombinant *Eh*CP1 protein was expressed in the cytosolic compartment of SHuffle Express cells harboring the pQEhCP1 plasmid. A fresh culture of transformed cells was sub-cultured (1:100) and grown at 37 °C for 2 h with shaking (300 rpm). Gene expression was induced by addition of IPTG to a final concentration of 0.5 mM. Expression was allowed by further growing at 30 °C for 18 h with shaking. Bacterial cells were harvested by centrifugation at 9300 x *g*, 10 °C for 10 min. Five cell pellets, each from a 200-mL batch, were obtained and preserved at − 20 °C.

Bacterial protein extracts were prepared by cell lysis under native conditions. Each pellet was suspended in 5 mL of TT-L buffer (1% Triton X-100; 100 mM Tris-HCl, pH 8.0; supplemented with 0.2 mg/mL lysozyme) and thoroughly mixed by rocking for 5 min. Cell lysis was achieved by sonication, using a typical procedure (10 cycles: 30 s ON, 30 s OFF; on ice bath), followed by rocking for 10 min. The cellular debris was removed by centrifugation at 9300 x g for 15 min at 10 °C. The supernatant (soluble fraction) was further cleared by centrifugation at 16000 x *g*, 10 °C for 15 min. Protein concentration was determined by conducting a Bradford assay [39], using BSA as the standard. Protease activity was estimated by conducting a chromogenic assay [40], using Z-Arg-Arg-pNA as the substrate.

### Purification of recombinant *Eh*CP1

Under native conditions, the recombinant *Eh*CP1 protein was purified from the cleared soluble fractions (CSF) of bacterial extracts by two consecutive standard chromatographic protocols: nickel-affinity and gel permeation. Initially diluted with one volume of BW buffer (300 mM NaCl; 100 mM Tris-HCl, pH 8.0; 20 mM imidazole, pH 8.0), CSF was then gradually loaded to a column containing nickel-nitrilotriacetic (Ni-NTA) agarose. After extensive washing, the bound protein was eluted with E buffer (300 mM NaCl; 100 mM Tris-HCl, pH 8.0; 250 mM imidazole, pH 8.0). Fractions containing a significant concentration of pure protein (> 95%) were pooled and slowly loaded to a PD-10 column (i.e., Sephadex^®^ G-25). Recombinant protein was eluted using 20 mM Tris–HCl buffer (pH 8.0). The complete purification process was monitored by SDS-PAGE [41] and gelatin zymography [42] analyses. The concentration and protease activity of recombinant *Eh*CP1 were measured as before.

### Protease assays using synthetic substrates

Enzymatic hydrolysis of Z-Arg-Arg-pNA (chromogenic) or Z-Arg-Arg-AMC (fluorogenic) was used to assess the protease activity of recombinant *Eh*CP1. The released product, p-nitroaniline (pNA) or 7-amino-4-methylcoumarine (AMC), was properly measured as a function of time to measure the proteolytic activity. Unless otherwise mentioned, standard assays (0.1 mL) were as follows:

(i) Chromogenic. Conducted at 37 °C, the enzyme (5 μg; i.e., 0.05 mg/mL) was initially activated for 10 min in citrate-phosphate buffer (50 mM; pH 6.5) containing 5 mM dithiothreitol (DTT). The reaction was started by adding the substrate (100 μM) to the assay mix. The linear increment of absorbance at 415 nm (A_415_) was determined from a 10-min kinetic analysis. Specific activity (SA) was expressed as units/mg (one unit is equal to 0.001 of A_415_ per min).

(ii) Fluorogenic. Performed at 22 ± 1 °C, the enzyme (0.5 μg; i.e., 0.005 mg/mL) was initially activated for 10 min in citrate-phosphate buffer (50 mM; pH 6.5) containing 5 mM DTT. The reaction was started by adding the substrate (5 μM) to the assay mix. The linear increment of fluorescence was determined from a 5-min kinetic analysis. Fluorescence was monitored at excitation and emission wavelengths of 355 and 460 nm. SA was expressed as FU/min/mg.

### Inhibition assay

The effect of E-64 on the protease activity of recombinant *Eh*CP1 was determined using different concentrations of the inhibitor (0 to 6 μM). The enzyme was initially incubated with the inhibitor for 15 min in reaction buffer without DTT. Then, this activator was added to the enzyme/inhibitor solution and after 10 min of additional incubation, the residual protease activity was measured as above, with a minor modification: 200 μM Z-Arg-Arg-pNA or 10 μM Z-Arg-Arg-AMC were used as the substrate.

### Enzyme and inhibition parameters

Km values were determined by linear least squares regression fitting of reciprocal values of the initial velocity against the substrate concentration according to the Lineweaver-Burk equation. IC_50_ values were established by non-linear least squares regression of the protease activity against the logarithm of the inhibitor concentration according to a dose-dependent variable-slope fitting curve. Both parameters were calculated using GraphPad Prism v. 4.0 for Windows (GraphPad Software, San Diego, CA, https://www.graphpad.com).

## Additional file

Additional file 1: Supplementary data. Schematic representations of the sequence coding for *Eh*CP1 (**Figure S1.**) and the recombinant plasmid pQEhCP1 (**Figure S2.**). Analysis of recombinant *Eh*CP1 purification (**Figure S3.**). (PDF 182 kb)

## Abbreviations

AMC: 7-Amino-4-Methylcoumarine
CP: Cysteine Protease(s)
E-64: L-Trans-3-Carboxyoxiran-2-Carbonyl-L-Leucylagmatine
*Eh*: *Entamoeba histolytica*
*Eh*CP1: Cysteine Protease 1 of *Entamoeba histolytica*
pNA: p-Nitroaniline
Z-Arg-Arg-AMC: Benzyloxycarbonyl-L-arginyl-L-arginine-7-amido-4-methylcoumarin
Z-Arg-Arg-pNA: Benzyloxycarbonyl-L-arginyl-L-arginine-p-nitroanilide
