# Supplementary material for: Soluble expression of an amebic cysteine protease in the cytoplasm of Escherichia coli SHuffle Express cells and purification of active enzyme
Source: BMC Biotechnol. 2018 Apr 3;18:20. doi: 10.1186/s12896-018-0429-y (PMC5883314; doi:10.1186/s12896-018-0429-y)
Supplement: Supplementary file 1 — Supplementary data. Schematic representations of the sequence coding for EhCP1 (Figure S1.) and the recombinant plasmid pQEhCP1 (Figure S2.). Analysis of recombinant EhCP1 purification (Figure S3.). (PDF 182 kb) [file 12896_2018_429_MOESM1_ESM.pdf]

BMC Biotechnology
Additional File 1. Supplementary Data

Methodology Article: Efficient soluble expression of an amebic cysteine protease in the cytoplasm of *Escherichia coli* SHuffle Express cells and purification of active enzyme

Ekaterina Jalomo-Khayrova [1], Rosa E. Mares [1], Patricia L. A. Muñoz [1], Samuel G. Meléndez-López [1], Ignacio A. Rivero [2], and Marco A. Ramos [1,*]

[1] Facultad de Ciencias Químicas e Ingeniería, Universidad Autónoma de Baja California, Calzada Universidad 14418, Parque Industrial Internacional; [2] Centro de Graduados e Investigación en Química, Instituto Tecnológico de Tijuana, Boulevard Industrial S/N, Mesa de Otay.
Tijuana, BCN 22510, México. *mramos@uabc.ecu.mx

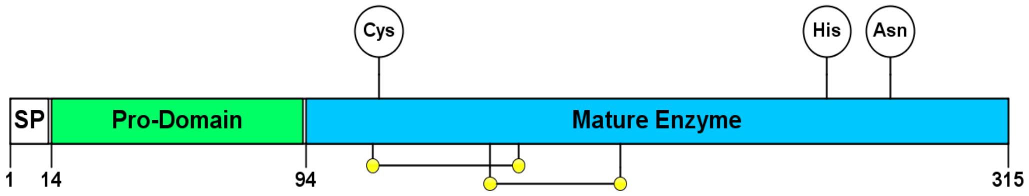

Figure S1. Schematic representation of the sequence coding for *Eh*CP1 (UniProtKB No. Q01957). Domain organization (amino to carboxy): signal peptide (SP, white), pro-domain (green), and mature enzyme (blue). Numbers denote the limits among them. The white circles show the relative position of the catalytic residues: Cys^{118}, His^{259}, and Asn^{279}. The yellow circles joined with a line display the predicted disulfide bonds (Cys^{115}-Cys^{161}; Cys^{152}-Cys^{193}).

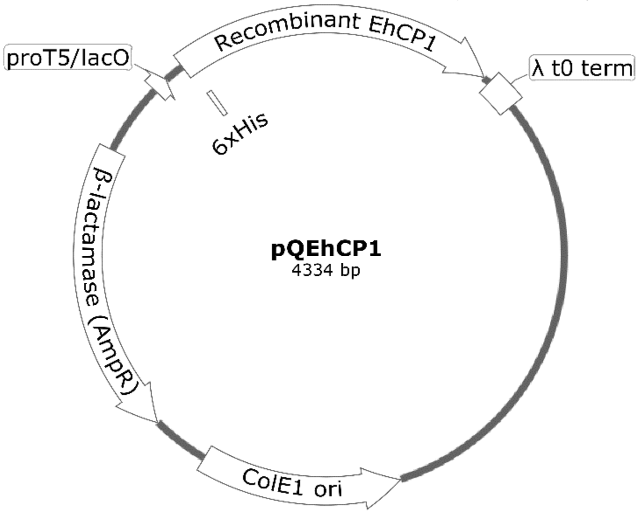

Figure S2. Schematic representation of the plasmid pQEhCP1 (4334 bp). A pQE30-derived plasmid having the ColE1 origin as autonomous replication sequence and the gene encoding the β-lactamase as selection marker. The gene sequence encoding the pro-mature *Eh*CP1 is located within proT5/lacO (promoter) and λ t0 term (terminator). The relative location of the hexahistidine tag (6xHis) is denoted as well.

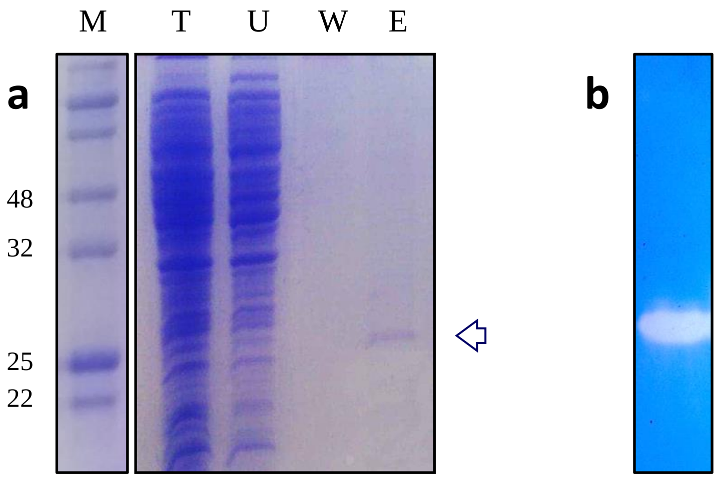

Figure S3. Analysis of recombinant *Eh*CP1 purification. (**a**) Electrophoretic separation of protein fractions obtained by nickel-affinity chromatography. 13.5% SDS-polyacrylamide gel stained with Coomassie Brilliant Blue. Lane, fraction: T, total soluble lysate; U, unbound; W, wash; E, elution. Lane M, molecular weight markers (New England Biolabs, Blue Protein Standard – Broad Range; kDa on the left). The arrow (on the right) indicates the relative mobility of recombinant *Eh*CP1. (**b**) Gelatin zymogram of activated recombinant *Eh*CP1. 13.5% SDS-polyacrylamide gel with 0.1% gelatin. After separation, the gel was treated as described [42]. Lane: 0.2 µg of elution fraction.

Reference

42. Serrano JJ, de la Garza M, Reyes M, León G, Tovar R, Muñoz ML *Entamoeba histolytica*: proteinase secretion induced by collagen type I is dependent on cytoskeleton integrity. Parasitol Res. 1996;82:200-5.
